# Association between intravenous iron therapy and short-term mortality risk in older patients undergoing hip fracture surgery: an observational study

**DOI:** 10.1186/s13018-021-02462-x

**Published:** 2021-05-18

**Authors:** Silas Zacharias Clemmensen, Kristian H. Kragholm, Dorte Melgaard, Lene T. Hansen, Johannes Riis, Christian Cavallius, Marianne M. Mørch, Maria Lukács Krogager

**Affiliations:** 1 Center for Clinical Research, North Denmark Regional Hospital, Hjørring, Denmark; 2 Department of Orthopedic Surgery, Aalborg University Hospital, Hjørring, Denmark; 3 Unit of Clinical Biostatistics and Epidemiology, Aalborg University Hospital, Aalborg, Denmark; 4 Department of Cardiology, Aalborg University Hospital, Aalborg, Denmark; 5 Department of Clinical Medicine, Aalborg University, Aalborg, Denmark; 6 Department of Geriatric Medicine, North Denmark Regional Hospital, Hjørring, Denmark; 7 Department of Emergency Medicine, Aalborg University Hospital, Aalborg, Denmark

**Keywords:** Hip fractures, Older people, Mortality, Anemia, Iron

## Abstract

**Background:**

Anemia is common among ortho-geriatric hip fracture patients and is associated with prolonged recovery and increased postoperative mortality rate. Intravenous iron seems to increase hemoglobin recovery and reduce the mortality rate in patients undergoing orthopedic surgeries. This study investigated the association between short-term mortality risk and intravenous iron therapy in older patients undergoing hip fracture surgery.

**Methods:**

This observational study included 210 patients undergoing hip fracture surgery from July 2018 to May 2020. These 210 patients were alive and had a hemoglobin ≤ 6.5 mmol/L on the 3rd postoperative day. In May 2019, a local intravenous iron therapy protocol was implemented and recommended intravenous iron (Monofer©) if hemoglobin on the 3rd postoperative day was ≤ 6.5 mmol/L. According to the treatment of postoperative anemia between the 1st and 3rd day post-surgery, the patients were divided into four groups: no treatment (*n*=52), blood transfusion (*n*=38), IV Monofer (*n*=80), and blood transfusion and IV Monofer (*n*=40). Primary outcome was 30-day mortality post-surgery. The secondary outcome was the impact on hemoglobin level 14–30 days postoperatively. Multivariable Cox regression was used to estimate the 30-day mortality standardized for covariates.

**Results:**

Of 210 patients, 17 (8.1%) died within 30 days after surgery. There was a significantly lower mortality among the patients who received IV Monofer compared to those who received no treatment (HR 0.17, 95% CI [0.03–0.93], *P* = 0.041). Among the 86 patients with available hemoglobin measurements within 14 to 30 days post-surgery, there was no significant difference in hemoglobin level between the various treatment groups (mean 6.6 mmol/L, *P* = 0.1165).

**Conclusion:**

IV Monofer on the 3rd postoperative day in older hip fracture patients seemed to reduce 30-day mortality compared with no treatment. No significant differences in hemoglobin levels between 14 and 30 days post-surgery across treatment groups were found, although this was assessed in a subset of patients with available hemoglobin levels warranting further study.

**Supplementary Information:**

The online version contains supplementary material available at 10.1186/s13018-021-02462-x.

## Background

Anemia is a severe complication among older people undergoing hip fracture surgery. Evidence suggests that anemia is associated with prolonged recovery, decreased mobility, and increased postoperative mortality rate [1–6]. Development of anemia is multifactorial: blood lost at the fracture site and during surgery, iatrogenic hemodilution, the presence of chronic diseases and inhibition of erythropoiesis, and functional iron deficiency due to trauma- and surgery-induced inflammation [7–9]. In the postoperative period, guidelines recommend treatment of severe anemia with allogenous blood transfusion (ABT). Besides heavy expense, ABT causes a higher risk of postoperative bacterial infections because of a immunological suppression, thus extending the hospitalization [10–12]. The alternative to ABT is stimulation of erythropoiesis with erythropoietin (EPO) and iron which seems to be associated with improved patient outcomes, reduced blood product utilization, reduced mortality, and product-related cost savings [13–22].

Several studies investigated the efficacy of intravenous (IV) iron administration perioperatively in patients undergoing major surgeries. On one hand, studies suggested that IV iron preoperatively is associated with reduced ABT rate, fewer nosocomial infections, decreased mortality, higher postoperative hemoglobin-concentration, and shorter hospitalization [12, 17, 19–21, 23–26]. On the other hand, a recent review concluded that the evidence for administration of IV iron preoperatively is deficient [27]. As for postoperative IV iron administration, the results are also contradictory. Kim et al. investigated the implementation of a strict transfusion protocol and postoperative IV iron among patients undergoing orthopedic hip surgery. They found that postoperative IV iron was related to a significantly lower number of transfused blood units pr. patient and higher hemoglobin-concentration 6 weeks postoperatively [28]. Contrarily, Moppett et al. have found no benefit of three daily doses of IV iron on the hemoglobin recovery 7 days postoperatively among older patients undergoing hip fracture surgery [29, 30].

As such, the evidence for administration of IV iron among older patients undergoing major orthopedic surgeries is scarce and conflicting. Therefore, in older patients undergoing hip fracture surgery, we wanted to investigate the impact of postoperative IV iron therapy on 30-day mortality and hemoglobin level 14–30 days postoperatively.

## Methods

### Study design and setting

This observational, single-center study of IV iron therapy included older patients admitted with an acute hip fracture at the Department of Orthopedic Surgery, Aalborg University Hospital, Hjørring, Denmark. In May 2019, a new local IV iron therapy protocol was implemented at the department and recommends IV iron isomaltoside 1000 (Monofer®) after surgery for hip fracture on the 3rd postoperative day if hemoglobin ≤ 6.5 mmol/L (≤ 10.4 g/dL) day three postoperatively. Therefore, the study investigates the time before (from July 2018 to May 2019) and after (August 2019 to May 2020) the implementation of the protocol.

### Outcome and follow-up

The primary outcome of the study is to analyze the 30-day mortality after surgery. The secondary outcome is to evaluate the efficacy of IV Monofer on the hemoglobin within 14 to 30 days postoperatively, although no systematic long-term follow-up on the hemoglobin was part of the IV iron therapy protocol.

### Study population

Patients admitted with an acute hip fracture at the Department of Orthopedic Surgery, Aalborg University Hospital, Hjørring, Denmark, from July 2018 to May 2019 (study period 1) and from August 2019 to May 2020 (study period 2) were included. Thus, the study period 1 was before the implementation of the iron treatment protocol, and IV iron was therefore administered non-systematically. In study period 2, IV iron was administered systematically to all patients with a hemoglobin ≤ 6.5 mmol/L on the 3rd postoperative day. This eligibility criterion of the iron therapy protocol is based on the evidence from major colorectal surgeries among patients diagnosed with colorectal cancer [31, 32]. Therefore, all patients with a hemoglobin > 6.5 mmol/L on the 3rd postoperative day were excluded. Furthermore, the patients who died in hospital before day three and the patients who were discharged immediately after surgery were excluded. Finally, the patients who fulfilled the eligibility criteria to IV iron but did not receive it because of patient denial, missing cooperation, or transfer to another hospital before day three was excluded.

### Study interventions

**Fig. 1:**
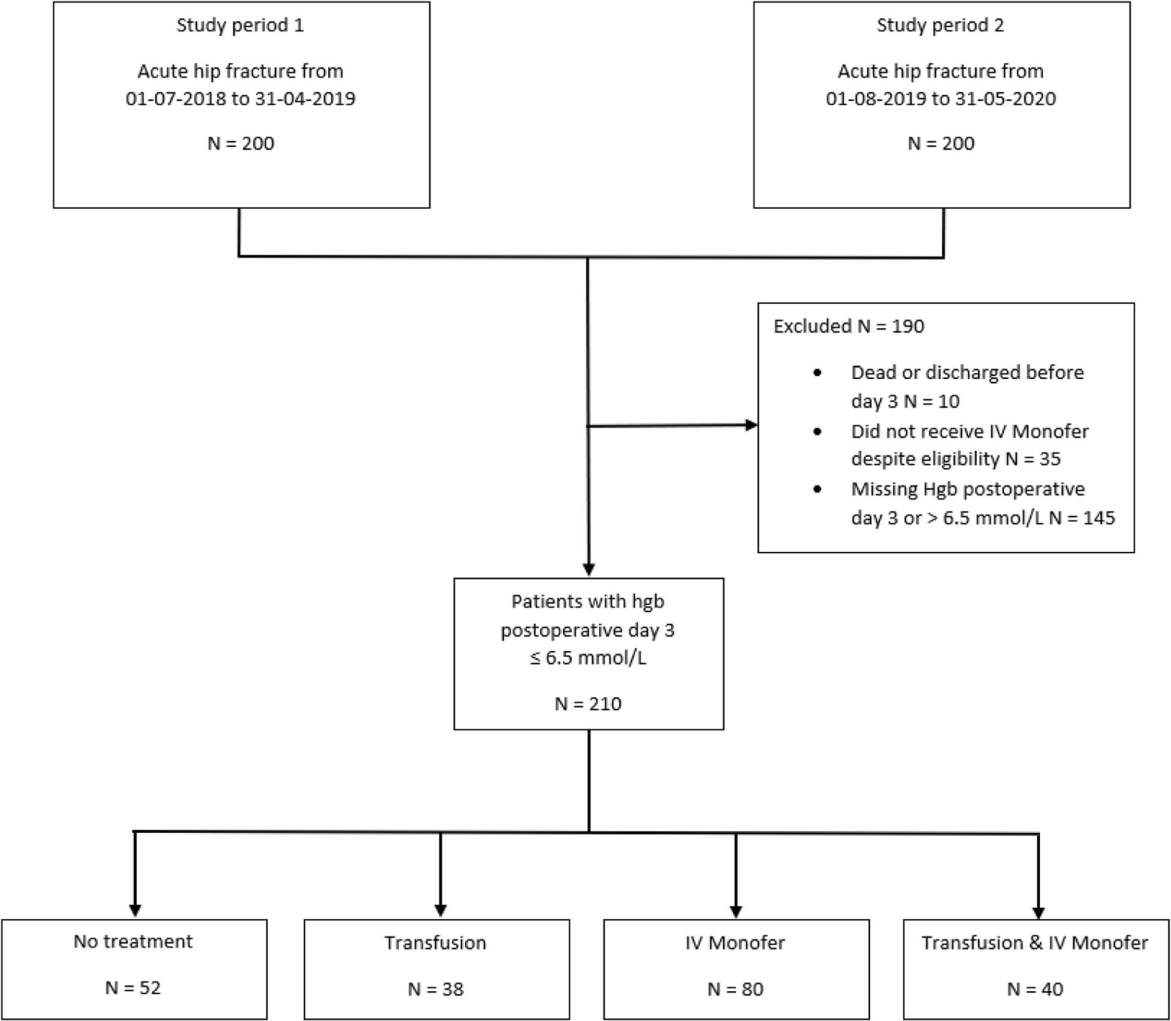
Study flowchart

An international consensus states postoperative anemia as hemoglobin < 6.83 mmol/L (< 11.0 g/dL) [16]. In this study, a hemoglobin ≤ 6.5 mmol/L on the 3rd postoperative day was defined as eligible according to the iron therapy protocol [33]. However, due to the non-systematic administration of IV iron in study period 1, IV iron was in this first period only prescribed to some patients after medical assessment by a geriatrician. Furthermore, if the postoperative anemia was severe, the treatment was initiated the 1st day after the surgery. After the implementation of the iron therapy protocol, IV iron was administered systematically. The patients with a hgb ≤ 6.5 mmol/L on the 3rd postoperative day were administered IV iron. The patients received a single dose of IV iron isomaltoside 1000 (Monofer®, Pharmacosmos) 20 mg/kg diluted in 100 mL isotone saline solution with an infusion time of 30 min while they were observed for adverse reactions [33, 34]. The indications for ABT followed the current local guidelines [35]. Therefore, the patients with a hemoglobin ≤ 6.5 mmol/L on the 3rd postoperative day were divided according to which treatment of postoperative anemia they received between day one and day three postoperatively. Accordingly, whether they received no treatment, IV iron, ABT, or both IV iron and ABT, see Fig. 1.

### Data collection

The data collection was performed in two steps and was obtained from the patients’ medical records retrospectively. Information was obtained on hospital admission date, hospital discharge date, number of readmissions, type of hip fracture, body mass index (BMI), 30 days mortality postoperatively, hgb at day one preoperatively, at day one and day three and between day 14 and 30 postoperatively, number of ABT, administration of IV Monofer, and concomitant comorbidities. The type of hip fracture was coded according to the World Health Organization International Classification of Disease (ICD), and the type of orthopedic procedure was classified according to Nomenclature for Properties and Units (NPU-codes). Secondly, we registered whether the patients were admitted from and discharged to home or nursing home and if they were readmitted to the hospital. Finally, we registered, date of surgery, reoperation during hospitalization, when IV Monofer, ABT, and antibiotics were administered, type of anesthesia and operation and perioperative blood loss.

### Comorbidities and concomitant medication

At hospitalization, we identified confounding factors such as comorbidities and concomitant medication. We consulted the national Shared Medication Record for information about the patients’ medication at submission time. Furthermore, we identified the occurrence of polypharmacy. Polypharmacy was defined as five or more medications according to a systematic review which found this to be the most commonly reported definition of polypharmacy [36].

Clinically relevant comorbidities were obtained from the patients’ medical records, classified according to the Charlson’s Comorbidity Index (CCI), and include previous myocardial infarction, congestive heart failure, peripheral vascular disease, cerebrovascular disease, dementia, chronic obstructive pulmonary disease, connective tissue disease, peptic ulcer disease, mild to severe liver disease, diabetes mellitus with or without complications, hemiplegia, moderate to severe chronic kidney disease, localized or metastatic solid tumor, leukemia, lymphoma, and AIDS [37, 38].

### Statistical analysis

Categorical variables were presented as counts and percentages, and continuous variables as median and 25th and 75th percentiles. The *χ*^2^ test was used to evaluate differences for categorical variables, and the Kruskal-Wallis test to evaluate differences for non-normally distributed continuous variables. Kaplan-Meier cumulative mortality curves were plotted for the four treatments of postoperative anemia to illustrate the crude 30-day mortality incidence. Using multivariable Cox regression and average treatment effect analysis (ATE), we reported the 30-day absolute and relative mortality risk standardized for the age, sex, medication, and comorbidity distribution of all patients eligible for analysis. A two-sided *p* value was considered statistically significant below 0.05. All data management and analyses were performed using and R, version 3.5.0 [39]..

## Results

### Patients

In total, 400 patients were recruited in the two study periods. After excluding patients not fulfilling the inclusion criteria, a total of 210 patients with hemoglobin ≤ 6.5 mmol/L on the 3rd postoperative day were included. We identified 52 patients with no treatment, 38 receiving ABT, 80 receiving IV Monofer, and 40 receiving IV Monofer and ABT.

### Characteristics

**Fig. 2:**
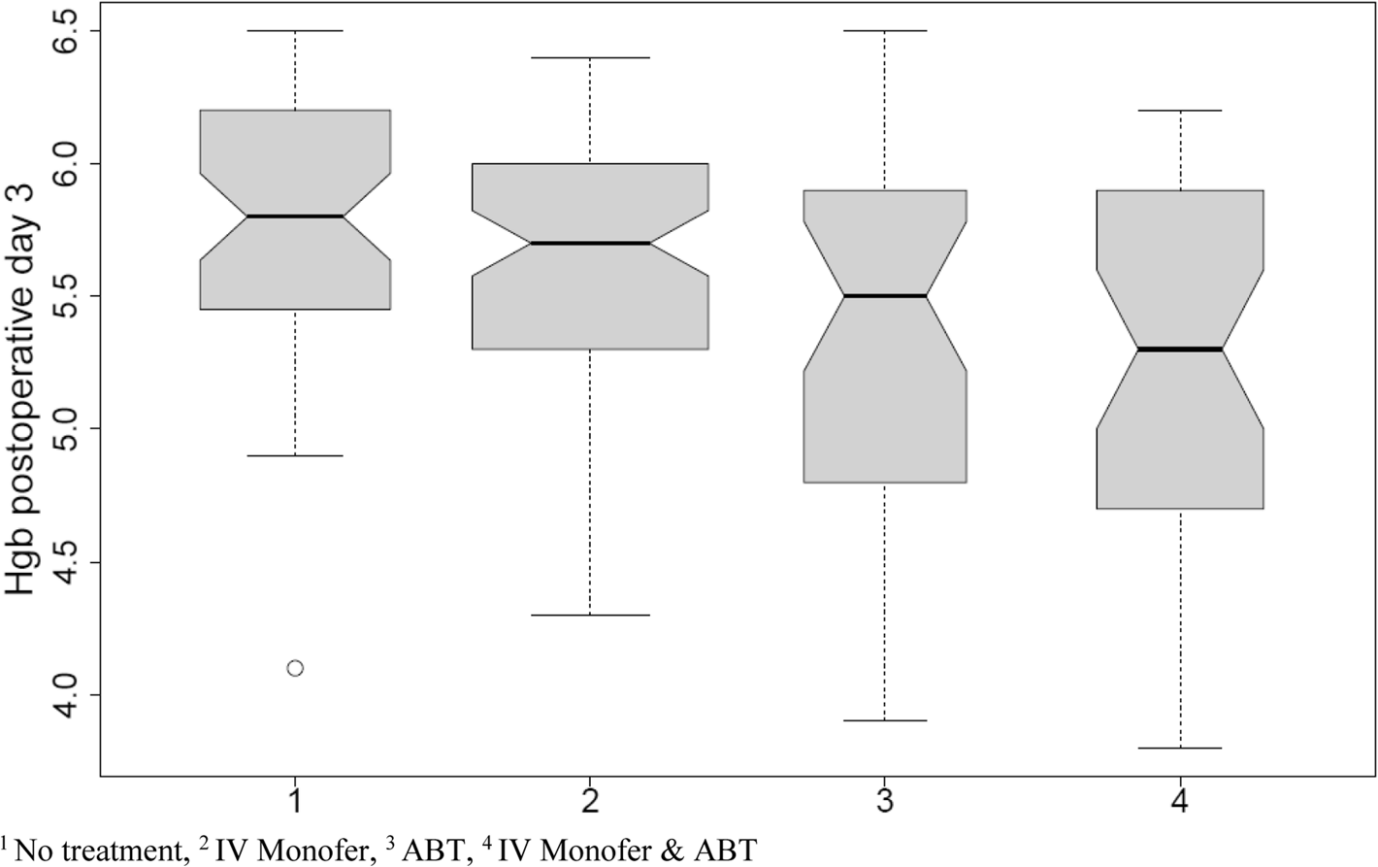
Hemoglobin level (mmol/L) on the 3rd postoperative day stratified by treatment groups. ^1^No treatment, ^2^IV Monofer, ^3^ABT, ^4^IV Monofer and ABT

The characteristics of the study population reported are presented in Table 1. The population consisted of 107 (51%) females and 103 (49%) males, and the average age was 83.5 (±8.7) years. There was no significant difference among the treatment groups regarding gender, age, BMI, CCI, admission source, and length of stay. However, patients in ABT and in the IV Monofer and ABT group had significantly more polypharmacy than patients in the other treatment groups. Furthermore, the patients receiving no treatment had significantly lower perioperative blood loss (185.7 ± 108.9 mL), fewer extracapsular fractures (44.2%), and fewer fixations with intramedullary nails (55.2%) than the other patients. Additionally, there was a significant difference in hemoglobin preoperatively and postoperatively among the treatment groups. The highest preoperative hemoglobin is 7.9 (±0.7) mmol/L in the IV Monofer group, and the lowest is 6.8 (±1) mmol/L in the IV Monofer and ABT group. The boxplot in Fig. 2 shows the distribution of the hemoglobin postoperative day three by the three possible treatment groups plus no treatment. Finally, a notable result was the significant change in choice of treatment in the two study periods, *p* < 0.0001. In study period 2, *N=*103, 101 patients (98%) received IV Monofer, 34 patients (33%) received ABT, and only 1 (1%) received no treatment. Contrarily, in study period 1, *N*=107, 19 patients (17.8%) received IV Monofer, 42 patients (39.3%) received ABT, and 51 patients (47.6%) received no treatment.

**Table 1:** Demographics among patients with hemoglobin ≤ 6.5 mmol/L on the 3rd postoperative day

	No treatment (*n*=52)	ABT (*n*=38)	IV Monofer (*n*=80)	IV Monofer and ABT (*n*=40)	Total (*n*=210)	*p* value
**Gender**						
Female	28 (53.8)	20 (52.6)	41 (51.2)	18 (45.0)	107 (51.0)	
Male	24 (46.2)	18 (47.4)	39 (48.8)	22 (55.0)	103 (49.0)	0.853
**Age**	82.6 (9.1)	83.2 (7.1)	83.6 (8.9)	84.7 (9.2)	83.5 (8.7)	0.709
**BMI (kg/m**^**2**^**)**	24.1 (4.2)	24 (5.1)	24.2 (3.9)	23.2 (3.8)	23.9 (4.2)	0.655
**Charlson Comorbidity Index**	1.9 (2)	2.7 (2.1)	1.9 (2.2)	2.1 (1.7)	2.1 (2.1)	0.219
**Polypharmacy (≥5 medications)**	34 (65.4)	33 (86.8)	55 (68.8)	34 (85.0)	156 (74.3)	0.023*
**Admission source**						
Home-independent	38 (73.1)	27 (71.1)	55 (68.8)	29 (72.5)	149 (71.0)	
Nursing home	14 (26.9)	11 (28.9)	25 (31.2)	11 (27.5)	61 (29.0)	0.951
**Study period**						
1	51 (98.1)	37 (97.4)	12 (15.0)	7 (17.5)	107 (51.0)	
2	1 (1.9)	1 (2.6)	68 (85.0)	33 (82.5)	103 (49.0)	< 0.001*
**Fracture**						
Intracapsular	29 (55.8)	14 (36.8)	31 (38.8)	13 (32.5)	87 (41.4)	
Extracapsular	23 (44.2)	24 (63.2)	49 (61.2)	27 (67.5)	123 (58.6)	0.098
**Operation**						
Arthroplasty	19 (36.5)	16 (42.1)	23 (28.8)	7 (17.5)	65 (31.0)	
Intramedullary nails	19 (36.5)	21 (55.3)	48 (60.0)	30 (75.0)	118 (56.2)	
AO screws	2 (3.8)	0 (0.0)	3 (3.8)	1 (2.5)	6 (2.9)	
Dynamic hip screws	7 (13.5)	1 (2.6)	4 (5.0)	1 (2.5)	13 (6.2)	
Other	5 (9.6)	0 (0.0)	2 (2.5)	1 (2.5)	8 (3.8)	0.014*
**Time to theatre (days)**	0.6 (0.5)	0.8 (0.8)	0.8 (0.5)	0.7 (0.6)	0.7 (0.6)	0.425
**Preoperative Hgb (mmol/L)**	7.7 (0.8)	7.4 (1)	7.9 (0.7)	6.8 (1)	7.5 (0.9)	< 0.001*
Missing	9	5	19	10	43	
**Perioperative blood loss (mL)**	185.7 (108.9)	387.1 (414.1)	205.1 (159.9)	254.7 (196.1)	246.8 (241.1)	0.001*
Missing	17	7	21	8	53	
**Hgb postoperative day 1 (mmol/L)**	6.3 (0.6)	5.4 (0.8)	6.1 (0.6)	5.1 (0.6)	5.8 (0.8)	< 0.001*
Missing	2	0	0	0	2	
**Hgb postoperative day 3 (mmol/L)**	5.8 (0.5)	5.4 (0.7)	5.6 (0.5)	5.2 (0.7)	5.6 (0.6)	< 0.001*
**Hgb postoperative between day** **14–30 (mmol/L)**	6.3 (0.7)	6.4 (0.5)	6.8 (0.6)	6.6 (0.8)	6.6 (0.7)	0.116
Missing because deceased	5	4	2	4	15	
Missing because no hgb control	28	21	48	12	109	
**Length of stay (days)**	6.5 (3.9)	7.1 (4)	5.9 (2.1)	6.9 (2.6)	6.5 (3.1)	0.177
**Discharged to**						
Home-independent	18 (34.6)	14 (36.8)	37 (46.2)	14 (35.0)	83 (39.5)	
Nursing home	34 (65.4)	20 (52.6)	43 (53.8)	24 (60.0)	121 (57.6)	
Dead in hospital	0 (0.0)	4 (10.5)	0 (0.0)	2 (5.0)	6 (2.9)	0.023*
**Readmission/transmission to ICU**^a^						
Readmitted	13 (25.0)	5 (13.2)	14 (17.5)	10 (25.0)	42 (20.0)	
Transmitted to ICU^a^	0 (0.0)	0 (0.0)	1 (1.2)	1 (2.5)	2 (1.0)	0.558

Data are presented as mean ± SD (age, BMI, Charlson Comorbidity Index, perioperative blood loss, time to theatre, hemoglobin levels, length of stay) or number of patients and percentage (all others)

^a^Intensive Care unit

*p < 0.05

### Mortality within 30 days after surgery

**Fig. 3:**
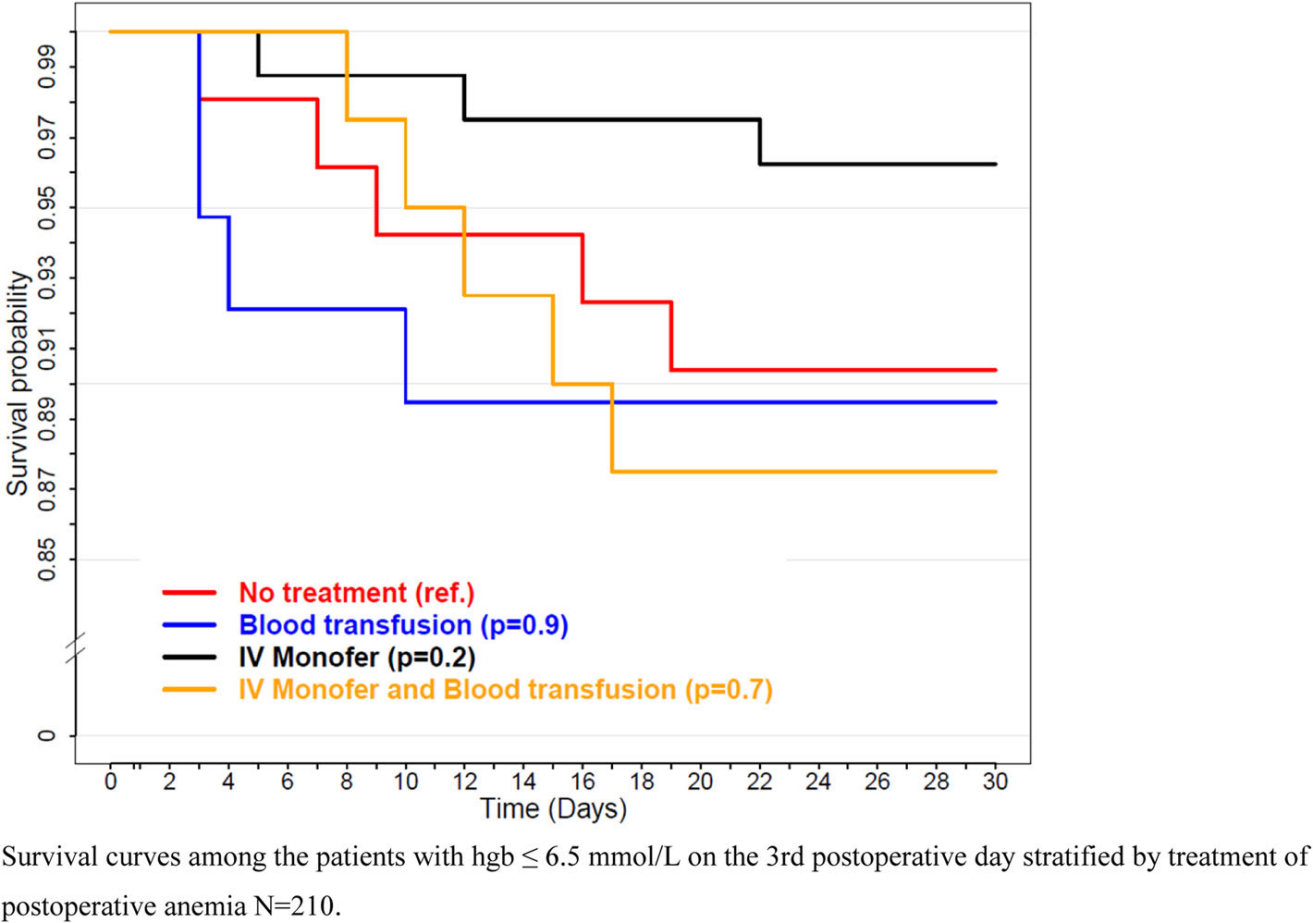
Kaplan-Meier curves. Survival curves among the patients with hgb ≤ 6.5 mmol/L on the 3rd postoperative day stratified by treatment of postoperative anemia *N*=210

In the population of 210 patients with hemoglobin ≤ 6.5 mmol/L on the 3rd postoperative day, 17 died during 30-day follow-up (8.1%). The main causes of death were age-related physical debility (52.9%) and a severe postoperative infection causing septic shock (23.5%). All other causes of death are reported in Additional file 1 Table S1. When stratified after treatment of postoperative anemia, the mortality was 9.6 (no treatment), 10.5 (ABT), 3.8 (IV Monofer), and 12.5% (IV Monofer and ABT), respectively. The survival curves are illustrated in Fig. 3. The results of the average treatment effect analysis and multivariable Cox regression model of the 210 patients stratified by treatment of postoperative anemia are shown in Table 2. The lowest mortality was observed in the IV Monofer treatment group (HR 0.41, 95% CI [0.089–1.887], *P* = 0.254). The average treatment effect analysis shows that the lowest standardized absolute mortality risk was observed in the two groups that received IV Monofer. Thus, the average risk in the IV Monofer and IV Monofer and ABT group was 2.31 and 9.5%, respectively. Additionally, the highest average mortality risk was observed in the no treatment and ABT group, with 18.49% and 17.77%, respectively. Hence, the IV Monofer group had an average mortality risk of 16.31% lower than the no treatment group and 15.46% lower than the ABT group.

**Table 2:** Estimation of the average treatment effect and multivariable Cox regression model among the patients with hgb postoperative day three ≤ 6.5 mmol/L stratified by treatment of postoperative anemia (30-day follow-up), *n*=210. The multivariable analysis is adjusted for age, gender, CCI, polypharmacy, admission source, and infection in hospital

ATE analysis			Multivariable Cox regression model		
	Average risk	CI (95%)	Hazard ratio	CI (95%)	*p* value
No treatment	0.184	[0.07–0.29]	1	Reference	
ABT	0.178	[0.06–0.30]	1.00	[0.245–4.087]	0.998
IV Monofer	0.023	[0.00–0.05]	0.41	[0.089–1.887]	0.254
IV Monofer and ABT	0.095	[0.03–0.16]	1.60	[0.399–6.434]	0.505

The results of the average treatment effect analysis and multivariable Cox regression when comparing the no treatment group with the IV Monofer group and the ABT group with the IV Monofer and ABT group, respectively, are presented in Tables 3 and 4. Administration of IV Monofer was associated with significantly decreased mortality risk compared to no treatment (HR 0.17, 95% CI [0.03–0.93], *P* = 0.041). However, the combination of IV Monofer and ABT showed a trend towards increased mortality compared to ABT alone (HR 1.34, 95% CI [0.269–6.653], *P* = 0.7215).

**Table 3:** Estimation of the average treatment effect and multivariable Cox regression model among patients with hgb postoperative day three ≤ 6.5 mmol/L stratified by no treatment and IV Monofer (30-day follow-up), *n*=132. The multivariable analysis is standardized for covariates

ATE analysis			Multivariable Cox regression model		
	Average risk	CI (95%)	Hazard ratio	CI (95%)	*p* value
No treatment	0.196	[0.027–0.364]	1	Reference	
IV Monofer	0.024	[0.000–0.051]	0.17	[0.03–0.93]	0.041*

**Table 4:** Estimation of the average treatment effect and multivariable Cox regression model among patients with hgb postoperative day three ≤ 6.5 mmol/L stratified by ABT and IV Monofer and ABT (30-day follow-up), *n*=78. The multivariable analysis is standardized for covariates

ATE analysis			Multivariable Cox regression model		
	Average risk	CI (95%)	Hazard ratio	CI (95%)	*p* value
ABT	0.220	[0.039–0.400]	1	Reference	
IV Monofer and ABT	0.072	[0.009–0.135]	1.34	[0.269–6.653]	0.7215

### Hemoglobin levels 14–30 days after surgery

When assessing the average hemoglobin postoperatively between day 14 and 30, a total of 124 (59%) patients was missing a hemoglobin level. Of the 124 patients missing hemoglobin leveld between 14 to 30 days after surgery, 15 (12%) were missing because they died before a new hemoglobin level was measured. The characteristics of these patients are reported in Additional file 1 Table S1. Furthermore, 109 (88%) patients had missing hemoglobin because it was not controlled at a general practitioner or at the geriatric outpatient clinic. Therefore, of the 210 patients, only 41% of them had their hemoglobin level controlled. Nevertheless, the patients treated with IV Monofer had the highest hemoglobin levels 14–30 days after surgery (6.8 ± 0.6 mmol/L) and the patients without treatment had the lowest (6.3 ± 0.7 mmol/L). Yet, no significant results between the four treatment groups were found.

## Discussion

This study analyzed the impact of a new IV iron therapy protocol among older patients with hip fracture and postoperative anemia according to 30-day mortality and hemoglobin levels 14–30 days postoperatively. The major finding was that the patients with hemoglobin ≤ 6.5 mmol/L on the 3rd postoperative day who only received IV Monofer had a significantly decreased risk of 30-day mortality compared to the patients with no treatment. The patients in the IV Monofer and the no treatment group had no significant difference in their comorbidity and pre- or postoperative hemoglobin level that could explain the decreased short-term mortality in the IV Monofer group. A plausible reason to the decreased short-term mortality is that 98.1% of the patients in the no treatment group was hospitalized in study period 1 before the implementation of the IV iron therapy protocol. Consequently, the patients with postoperative anemia in study period 1 without indication of ABT were not treated with IV Monofer. Therefore, our data suggested that the patients with hemoglobin below 6.5 mmol/L but above transfusion limit might benefit from IV Monofer.

In general, few studies have reported the postoperative mortality after perioperative IV or oral iron therapy among patients undergoing major orthopedic surgical procedures [20, 23, 28, 29, 40–43]. A retrospective analysis of 2547 patients after orthopedic surgery reported a mortality of 4.8% in 1000 patients receiving either iron sucrose (Venofer) or iron sucrose + recombinant human EPO perioperatively, against 9.4% in the control population of 361 patients [23]. Likewise, Cuenca et al. found that iron sucrose preoperatively in elderly patients undergoing displaced subcapital hip fracture repair (*n*=20) compared to a control group (*n*=57) had a lower 30-day mortality rate (0 vs. 19.3%) [20]. However, as stated in the systematic review by Smith et al., the evidence for preoperative IV iron therapy in improving clinical outcomes in patients undergoing major orthopedic surgery is little. Nevertheless, our findings concur with these previous studies regarding a significant reduction in postoperative mortality although our IV iron therapy was in the postoperative phase.

Furthermore, we reported the average correction of the postoperative anemia. The increase in postoperative hemoglobin after the treatment with either ABT and/or IV Monofer was based on the first measured hemoglobin level within 14–30 days postoperatively. In summary, we found no significant difference in hemoglobin between the various treatment options. However, we acknowledge that the missing data on the patients’ hemoglobin level between 14 and 30 days postoperatively was affecting the results. Thus, the patients who had hemoglobin measured between 14 and 30 days after surgery are probably not representative of the exact hemoglobin in this postoperative period of all the patients in our study. Nevertheless, when comparing our results with other studies, we acknowledge that the results on whether perioperative IV iron significantly recovers the hgb postoperatively are conflicting [24, 28, 29, 42–45]. Johansson et al. explored whether Monofer results in a better regeneration of hemoglobin-concentration and prevented anemia compared to placebo in preoperative non-anemic patients undergoing cardiac surgery. They showed that a single, perioperative 1000-mg dose of Monofer significantly increased the hemoglobin-concentration (mean 1.18 mmol/L) and prevented anemia 4 weeks after surgery [24]. Contrary, the study by Moppett et al. among older people with hip fracture showed that three doses of 200 mg Venofer on three separate days had no remarkable effect on final hemoglobin-concentration on the 7th day postoperatively [29]. Additionally, we acknowledge that the hemoglobin recovery especially depends on when the hemoglobin is measured postoperatively as studies have shown that IV Monofer requires several weeks before the physiological effect on the hemoglobin level is measurable [24, 44]. Nevertheless, no such systematic long-term follow-up on the hemoglobin was part of the present IV iron therapy protocol in our study. Therefore, further prospective and randomized controlled trials examining the efficacy of postoperative IV Monofer therapy regarding hemoglobin recovery among older hip fracture patients are required.

### Strength and limitations

A strength of the study is the availability of information and the registration of clinical variables from the patients’ medical records and the national Shared Medication Record. Furthermore, the study shows that the implementation of the IV iron therapy protocol in the clinical setting caused a change in choice of treatment since a large percentage of the patients in study period 2 received IV Monofer. However, our study had several limitations. The observational nature of the study implies only associations therefore no causal relations can be concluded. First, the risk of confounding is related to the nature of the study. Therefore, in the survival analysis, we applied standardization to ensure that the study population had similar age, gender, comorbidity, polypharmacy, admission source, and hospital infection status to evaluate the impact of IV Monofer on outcomes. Second, we acknowledge that the size of the study population affected the statistical power. Third, we acknowledge that undergoing a hip fracture surgery is associated with an increased risk of postoperative pneumonia and pneumothorax due to intubation during the general anesthesia, time of operation, and immobilization the first day postoperatively. Therefore, data regarding SpO2, arterial blood gas, and chest X-ray would have been relevant to assess whether the patients had a respiratory complication which affected the recovery and the mortality of the patients. Finally, 59% of the patients did not have available hemoglobin measurements after discharge from the hospital as patients were not systematically followed up with blood tests at their general practitioner or at the geriatrician ambulatory. Therefore, the patients who had hemoglobin measured in the 14-30-day span after surgery are likely not representative of the true hemoglobin distribution of all patients, if all patients had a hemoglobin measured in this period. In other words, the hemoglobin measured was driven by a clinical contact and thus, conclusions regarding the efficacy of Monofer in increasing the hemoglobin level postoperatively cannot adequately be made from our data.

## Conclusion

In conclusion, the patients with hemoglobin ≤ 6.5 mmol/L on the 3rd postoperative day who only received IV Monofer had a significantly decreased risk of 30-day mortality compared to the patients with no treatment. Furthermore, no significant differences in hemoglobin levels between 14 and 30 days post-surgery across treatment groups were found, although this was assessed in a subset of patients with available hemoglobin levels.

## Supplementary Information

**Additional file 1: Table S1.** Causes of death among the patients who died within 30-days postoperatively. **Table S2.** Demographics of patients who died before hemoglobin level between day 14 to 30 was measured.

## Data Availability

The datasets used and/or analyzed during the current study are available from the corresponding author on reasonable request.

## Abbreviations

IV: Intravenous
EPO: Erythropoietin
ABT: Allogenous blood transfusion
CCI: Charlson Comorbidity Index
BMI: Body mass index
ICU: Intensive care unit
ICD: International Classification of Disease
NPU: Nomenclature for Properties and Units
ATE: Average treatment effect analysis
