# Supplementary material for: Association between intravenous iron therapy and short-term mortality risk in older patients undergoing hip fracture surgery: an observational study
Source: J Orthop Surg Res. 2021 May 18;16:320. doi: 10.1186/s13018-021-02462-x (PMC8130414; doi:10.1186/s13018-021-02462-x)
Supplement: Supplementary file 1 — Additional file 1: Table S1. Causes of death among the patients who died within 30-days postoperatively. Table S2. Demographics of patients who died before hemoglobin level between day 14 to 30 was measured. [file 13018_2021_2462_MOESM1_ESM.docx]

**Table 1 causes of death among the patients who died within 30-days postoperatively.**

|  | **No treatment (n=5)** | **ABT (n=4)** | **IV Monofer (n=3)** | **IV Monofer & ABT (n=5)** | **Total (n=17)** | **p-value** |
| --- | --- | --- | --- | --- | --- | --- |
| Septic shock  (DR65.21) | 0 (0.0) | 2 (50.0) | 1 (33.3) | 1 (20.0) | 4 (23.5) |  |
| Acute myocardial infarction  (DI21.9) | 1 (20.0) | 1 (25.0) | 0 (0.0) | 0 (0.0) | 2 (11.8) |  |
| Pulmonary edema  (DJ81.0) | 0 (0.0) | 0 (0.0) | 0 (0.0) | 1 (20.0) | 1 (5.9) |  |
| Pneumonia  (DJ18.9) | 0 (0.0) | 0 (0.0) | 0 (0.0) | 1 (20.0) | 1 (5.9) |  |
| Age-related physical debility  (DR54) | 4 (80.0) | 1 (25.0) | 2 (66.7) | 2 (40.0) | 9 (52.9) | 0.166 |

Data are presented as number of patients and percentage. Death causes are presented with the Classification of Diseases (ICD-10) code.

**Table 2. Demographics of patients who died before hemoglobin level between day 14 to 30 was measured**

|  | **No treatment (n=5)** | **ABT (n=4)** | **IV Monofer (n=2)** | **IV Monofer & ABT (n=4)** | **Total (n=15)** | **p-value** |
| --- | --- | --- | --- | --- | --- | --- |
| **Gender** |  |  |  |  |  |  |
| Female | 4 (80.0) | 4 (100.0) | 1 (50.0) | 2 (50.0) | 11 (73.3) |  |
| Male | 1 (20.0) | 0 (0.0) | 1 (50.0) | 2 (50.0) | 4 (26.7) | 0.356 |
| **Age** | 84.7 (8.5) | 84.9 (6.6) | 89.6 (6.4) | 88 (1.2) | 86.3 (6.1) | 0.741 |
| **BMI (kg/m^2^)** | 20.4 (4) | 26 (5.1) | 19.5 (1.8) | 25.2 (5.3) | 23.5 (5) | 0.213 |
| **Charlson Comorbidity Index** | 2.4 (1.1) | 3.8 (0.5) | 7.5 (3.5) | 3 (1.4) | 3.6 (2.1) | 0.005* |
| **Polypharmacy**  **(≥5 medications)** | 3 (60.0) | 4 (100.0) | 2 (100.0) | 4 (100.0) | 13 (86.7) | 0.202 |
| **Admission source** |  |  |  |  |  |  |
| Home-independent | 2 (40.0) | 2 (50.0) | (0.0) | 1 (25.0) | 5 (33.3) |  |
| Nursing home | 2 (60.0) | 2 (50.0) | 2 (100.0) | 3 (75.0) | 10 (66.7) | 0.631 |
| **Study period** |  |  |  |  |  |  |
| 1) | 5 (100.0) | 4 (100.0) | 0 (0.0) | 0 (0.0) | 9 (60.0) |  |
| 2) | 0 (0.0) | 0 (0.0) | 2 (100.0) | 4 (100.0) | 6 (40.0) | 0.002* |
| **Fracture** |  |  |  |  |  |  |
| Intracapsular | 3 (60.0) | 2 (50.0) | 0 (0.0) | 3 (75.0) | 8 (53.3) |  |
| Extracapsular | 2 (40.0) | 2 (50.0) | 2 (100.0) | 1 (25.0) | 7 (46.7) | 0.369 |
| **Operation** |  |  |  |  |  |  |
| Arthroplasty | 3 (60.0) | 3 (75.0) | 0 (0.0) | 1 (25.0) | 7 (46.7) |  |
| Intramedullary nails | 1 (20.0) | 1 (25.0) | 2 (100.0) | 2 (50.0) | 6 (40.0) |  |
| AO screws | 0 (0.0) | 0 (0.0 | 0 (0.0) | 0 (0.0) | 0 (0.0) |  |
| Dynamic hip screws | 1 (20.0) | 0 (0.0) | 0 (0.0) | 1 (25.0) | 2 (13.3) |  |
| other | 0 (0.0) | 0 (0.0) | 0 (0.0) | 0 | 0 (0.0) | P = NA |
| **Time to theatre (days)** | 0.4 (0.6) | 1 (0.0) | 0.5 (0.5) | 0.7 (0.3) | 0.6 (0.4) | 0.264 |
| **Preoperative Hgb (mmol/L)** | 8.1 (0.7) | 7.2 (0.5) | 7.9 (0.2) | 5.6 (0.8) | 7.2 (1.1) | < 0.001* |
| Missing | 1 | 0 | 0 | 1 | 2 |  |
| **Perioperative blood loss (mL)** | 133.3 (104.1) | 325 (263) | 50 | 150 (70.7) | 195.8 (179) | 0.315 |
| Missing | 2 | 0 | 1 | 0 | 3 |  |
| **Hgb postoperative day 1 (mmol/L)** | 6.9 (0.4) | 5 (0.7) | 6 (0.6) | 5.3 (0.6) | 5.9 (1) | < 0.001* |
| **Hgb postoperative day 3 (mmol/L)** | 6 (0.6) | 5.3 (0.7) | 5.6 (0.5) | 5.5 (0.5) | 5.6 (0.6) | 0.415 |
| **Length of stay (days)** | 3.6 (2.1) | 5.5 (3.7) | 6 (1.4) | 5 (1.8) | 4.8 (2.4) | 0.591 |
| **Discharged to** |  |  |  |  |  |  |
| Home-independent | 1 (20.0) | 0 (0.0) | 0 (0.0) | 0 (0.0) | 1 (6.7) |  |
| Nursing home | 4 (80.0) | 0 (0.0) | 2 (100.0) | 3 (75.0) | 9 (60.0) |  |
| Dead in hospital | 0 (0.0) | 4 (100.0) | 0 (0.0) | 1 (25.0) | 5 (33.3) | 0.040* |
| **Readmission/transmission to ICU^$^** |  |  |  |  |  |  |
| Readmitted | 2 (40.0) | 0 (0.0) | 1 (50.0) | 0 (0.0) | 3 (20.0) |  |
| Transmitted to ICU^$^ | 0 (0.0) | 0 (0.0) | 0 (0.0) | 0 (0.0) | 0 (0.0) | P = NA |

Data are presented as mean ± SD (age, BMI, Charlson Comorbidity Index, perioperative blood loss, time to theatre, hemoglobin levels, length of stay) or number of patients and percentage (all others). ^$^Intensive Care unit.
